# Evaluation of Salivary Matrix Metalloproteinase (MMP-8) in Periodontal Patients Undergoing Non-Surgical Periodontal Therapy and Mouthwash Based on Ozonated Olive Oil: A Randomized Clinical Trial

**DOI:** 10.3390/ijerph17186619

**Published:** 2020-09-11

**Authors:** Gianna Maria Nardi, Francesca Cesarano, Giulio Papa, Lorella Chiavistelli, Roman Ardan, Maciej Jedlinski, Marta Mazur, Roberta Grassi, Felice Roberto Grassi

**Affiliations:** 1 Department of Dental and Maxillofacial Sciences, Sapienza University of Rome, 00161 Rome, Italy; profnardi.giannamaria@gmail.com (G.M.N.); francesca.cesarano88@gmail.com (F.C.); gpapa82@gmail.com (G.P.); lorella.chiavistelli@gmail.com (L.C.); 2 Chair of Econometrics, Department of Economic Sciences, Koszalin University of Technology, 75-343 Koszalin, Poland; roman.ardan@tu.koszalin.pl; 3 Department of Interdisciplinary Dentistry, Pomeranian Medical University in Szczecin, 70-111 Szczecin, Poland; maciej.jedlinski@hotmail.com; 4 Department of Biomedical Sciences, University of Sassari, 07100 Sassari, Italy; grassi.roberta93@gmail.com; 5 Department of Basic Medical Sciences, Neurosciences and Sense Organs, Aldo Moro University of Bari, 70122 Bari, Italy; feliceroberto.grassi@uniba.it

**Keywords:** ozonated oil, periodontitis, salivary metalloproteinase, RCT, olive oil, ozone

## Abstract

*Background*: Extracellular matrix metalloproteinases (MMPs) play a pivotal role in the damage to the periodontal tissue in patients with periodontitis. Scaling and root planning (SRP) attempt to control the plaque amount and consequentially reduce the bacterial load. Non-surgical periodontal treatment could be integrated with drug therapy and physiotherapy procedures such as ozone therapy. The aim of this study was to evaluate in a cohort of patients with a diagnosis of periodontitis: (1) the efficacy of non-surgical periodontal therapy assisted by the use of ozonated olive oil-based mouthwash on salivary metalloproteinase (MMP-8) and (2) the reduction of periodontal indices. *Methods*: Ninety-six subjects with a diagnosis of periodontitis were enrolled in this study and randomly assigned to the study group (SRP + mouthwash) or control group (SRP). The study duration was 3 months. Data on MMP-8, plaque index (PI), bleeding on probing (BoP) and probing pocket depth (PPD) were recorded at T0, T1 (14 days), T2 (1 month) and T3 (6 months). Group differences were assessed using Student’s *t*-test for independent samples. *Results:* A significant improvement in PI, BoP, PPD and salivary MMP-8 levels was observed in both groups. An analysis of differences in relative changes of indices revealed the efficacy of ozonated olive oil in decreasing MMP-8 level. Simultaneously, it slowed the decrease of BoP index. *Conclusions:* Scaling and root plaining with the aid of ozonated olive oil mouthwash were found to be more effective on salivary MMP-8 reduction than scaling and root plaining alone.

## 1. Introduction

The activation of the cells of the immune system induces the release of enzymes responsible for damage to connective tissue and the consequent destruction of periodontal tissue. A crucial role in these processes has been attributed to extracellular matrix metalloproteinases (MMPs).

MMPs belong to a large group of metal-dependent endopeptidases (Zn2+) synthesized from connective tissue cells [1,2,3]. The matrix metalloproteinases are secreted as latent proenzymes and activated in the extracellular environment. The activation consists of breaking the bond between Zn and cysteine, which causes the blocking of enzymatic reactivity [1,2,4].

MMP-8 (neutrophilic collagenase; collagenase-2) is assembled into specific granules of polymorphonuclear leukocytes (PMNs). Its presence has also been detected in human chondroblasts, and gingival and periodontal fibroblasts [5,6,7,8,9]. MMP-8 is responsible for the destruction of type I, II and III collagen [3,10,11]. Ingman et al. [10] and Golub et al. [11] were the first to demonstrate that MMP-8 is the main enzyme present in situ in the gingival tissue and salivary fluid, and most likely initiates the destruction of the site’s tissues with active inflammation.

The primary goal of the periodontal treatment is to decrease the amount of plaque and to control the bacterial load in order to stop inflammation and arrest disease progression. Non-surgical periodontal therapy (scaling and root planning (SRP)) with the adjunctive use of local chemotherapeutic agent is the first treatment of choice, due to its limited invasiveness. Chlorhexidine is the gold standard among the existing local antiseptic agents, but it showed several side effects when used over time (tooth discoloration, taste alterations and oral desquamation) and the clinical outcomes may be hampered by lack of patient compliance [12,13,14].

Therefore, new antimicrobial agents are needed to perform SRP in patients with periodontitis, with good biocompatibility and without limitations due to side effects correlated with long-term use. Currently, ozone therapy in dentistry is a modern non-invasive treatment, with antimicrobial, immunostimulating and acceleration of the wound healing rate effects. It can be used as ozonated water and ozonated olive oil. Studies in the field of periodontology documented their effectiveness against both Gram-positive and Gram-negative bacteria, viruses and fungi [15].

The primary and secondary aims of the present study were to prospectively evaluate in a cohort of patients with a diagnosis of periodontitis: (1) the efficacy of non-surgical periodontal therapy assisted by the use of ozonated olive oil-based mouthwash on salivary metalloproteinase (MMP-8) values and (2) the reduction of periodontal indices.

## 2. Materials and Methods

The protocol was approved by the local Ethical Committee (approval number 0399) and informed consent was obtained from all individuals. All the procedures were in accordance with the 1964 Helsinki Declaration and its later amendments or comparable ethical standards.

### 2.1. Study Design

A randomized, double-blind, case-control study was conducted. The analysis included data from all patients with different stages of periodontitis. After enrollment, the patients were divided into two groups, according to randomization: study and control.

### 2.2. Study Population and Setting

Ninety-six subjects of both sexes, aged between 30 and 60 years, were selected by Dr. Lorella Chiavistelli (L.C.) at a private dental practice in Cecina, Italy. The enrolled subjects were subsequently divided into two groups: control group (*n* = 48, scaling and root planning procedures) and experimental group (*n* = 48, scaling and root planning procedures + mouthwash based on ozonated olive oil).

### 2.3. Sample Size Calculation

Without previous data available in the literature, a prevalence of 0.5 was considered. The confidence interval was set at 95%, with precision 1, to the extent that a margin of error of 10% was acceptable. Given these variables, 96 patients were required. By reducing the margin of error and increasing the accuracy, the sample number would increase according to the usual parameters.

### 2.4. Inclusion Criteria

1. Male or female, 30–60 years old;
2. With diagnosis of periodontal disease;
3. Plaque index (PI) ≥ 35%, Gingival index (GI) ≥ 35%;
4. No diabetes and cardiovascular diseases;
5. No pharmacological therapies;
6. No smoking, alcohol and/or drug consumption;
7. No pregnancy or breastfeeding;
8. No allergy.

### 2.5. Randomized Allocation

The enrolled patients were randomly assigned to the study or control group. A randomized allocation was carried out as follows: the group was chosen according to the patient’s birth date. Odd numbers were allocated to the study group (SG), while even numbers were allocated to the control group (CG).

### 2.6. Blinding

Blinding of data collectors and outcome adjudicators was achieved.

### 2.7. Periodontal Charting

A blind operator to the allocation procedure, Prof. Gianna Maria Nardi (GM.N.), completed the periodontal chart at T0.

Probing pocket depth (PPD), plaque index (PI) and bleeding on probing (BoP) were assessed and recorded.

### 2.8. Salivary MMP-8 Assessment

Salivary active matrix metalloproteinase levels were analyzed by PerioSafe® (Dentagnostics, Jena, Germany), a diagnostic test for the prevention of periodontal and peri-implant disease. The test shows the high risk of progressive degeneration of the periodontium. The assessments were performed at T0, T1, T2 and T3.

### 2.9. Description of the Mouthwash

Patients in the SG received indications to the use of the mouthwash based on ozonated olive oil (Ialozon Blu, Gemavip, Italy) for 3 months (12 mL, rinses of 30 s, 3 times a day).

### 2.10. Clinical Procedure

At T0, T1, T2 and T3:

Periodontal chart parameters (PI, BoP and PPD) were detected using the PCP 15-mm periodontal probe (Hu-Friedy, periodontal probe graduated at intervals of millimeters, bold marks at 5 mm, 10 mm and 15 mm).

Deplaquing was carried out with air polishing, with the 120° insert and glycine powder; subsequently, the 90° insert with bicarbonate powder was used to remove the most important extrinsic pigmentations above the gums (Mectron Combi Touch, Mectron Spa, Carasco (GE), Italy).

Scaling with S1 insert and scaling under the gingival with P10 universal periodontal insert were performed; in active sites with probing ≥ 5 mm, a disposable sterile perio tip and glycine powder were added.

### 2.11. Co-Intervention

The oral care of the enrolled patients was standardized: the same toothpaste (Sensodyne Rapid Action, GlaxoSmithKline, SmithKline Beecham Ltd., Slough, UK), toothbrush (GUM^®^ Technique^®^ PRO toothbrush, medium bristles, Sunstar Europe, Etoy, Switzerland) and interproximal cleaner (GUM Soft-Picks^®^ Advanced, Sunstar Europe, Etoy, Switzerland) were delivered to all subjects. Patients received instructions for the at-home oral hygiene procedures based on the Tailored Brushing Method (TBM) [16].

### 2.12. Duration of the Study and Times of Follow-Up

The study period was 6 months. Patients were treated at baseline (T0) and at 14 days (T1), 1 month (T2) and 3 months (T3). Periodontal indices were recorded at all times of follow-up and a database was collected by Dr. Roberta Grassi (R.G.).

### 2.13. Statistical Analysis

The study population was analyzed by the intention-to-treat (ITT) principle, and then all randomized patient data were analyzed. Frequencies, means, medians and standard deviation were calculated for the descriptive analysis. The efficacy of treatment in both groups and between-group differences were assessed using the Student’s *t*-test for independent samples. The results were considered to be statistically significant at *p* < 0.05. The R statistical package (The R Foundation for Statistical Computing, Wirtschaftsuniversität Wien, Vienna, Austria) was used for the calculations.

## 3. Results

**Figure 1:**
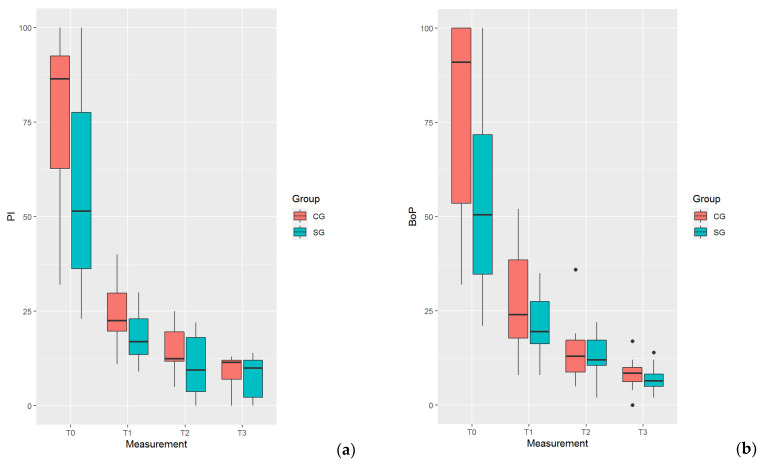

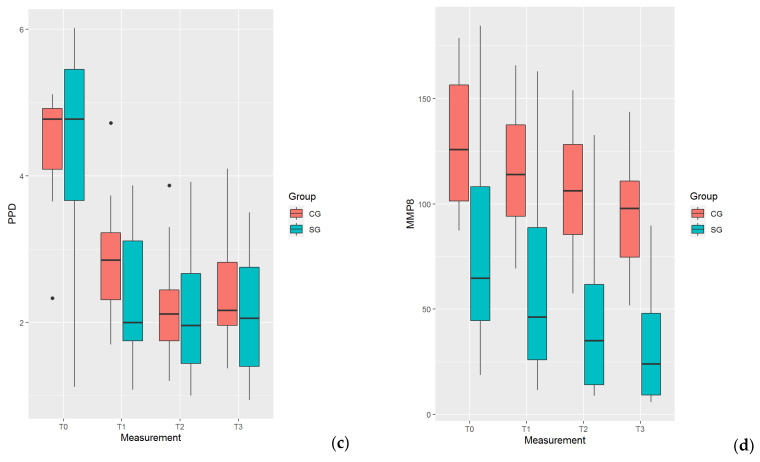
Changes in the distribution of periodontal indices (**a**–**c**) and salivary metalloproteinase (MMP-8) levels (**d**).

Changes in the distribution of periodontal indices and salivary MMP-8 levels during treatment in both groups are presented in Figure 1a–d and Table 1.

Groups were significantly different in means at baseline (*p* < 0.001), except for the PPD index. Groups had significantly different variances for PPD and MMP-8 indices (*p* < 0.001 in Levene’s test).

The decrease in indices was calculated at T1, T2 and T3 comparing with baseline in order to compare the efficacy of both treatments. Taking into account baseline differences, relative decreases were considered.

$$Relative\;decrease=\frac{baseline\;value-current\;value}{baseline\;value}$$

Relative decrease was in the [0; 1] interval (assuming index decrease) and showed what part of initial (baseline) value of the index was eliminated till moments T1, T2 and T3. Changes of all indices at T1, T2 and T3 were statistically significant (*p* < 0.001). A comparison of the groups is presented in Table 2.

An analysis of differences in the relative reduction of indices revealed the efficacy of ozonated olive oil in reducing the MMP-8 level. Simultaneously, it slowed the decrease of BoP index, and, at the beginning of treatment (at T1), of PI index as well. Between-group differences in PPD and PI at T2 and T3 were insignificant.

## 4. Discussion

This study assessed for the first time the levels of salivary metalloproteinase MMP-8 in periodontal patients undergoing non-surgical periodontal therapy assisted by the use of mouthwash based on ozonated olive oil. The results showed that non-surgical periodontal treatment with the aid of the ozonated olive oil mouthwash was more effective on salivary MMP-8 reduction than non-surgical treatment alone.

The secondary aim of the present study was to evaluate the reduction of periodontal indices. The results showed no significative difference between SG and CG in reducing the periodontal indices.

Several trials are present in literature on the effect of ozone in periodontal treatment. Brauner [17] compared the clinical periodontal status between patients undergoing SPR and patients trained to use only ozonated water for oral rinses, concluding that the latter cannot replace professional removal of dental plaque. However, the use of ozone can decrease the inflammatory state. Menable et al. [18] demonstrated the therapeutic effect of ozone in the treatment of periodontitis, with reduction in GI, PI and PPD.

Many authors have reported a decrease in salivary MMP-8 after conventional periodontal therapy [19,20,21,22,23,24,25]. The current study showed that patients treated exclusively with SRP showed a less significant decrease in MMP-8 than patients treated with SRP with the aid of an ozonated olive oil mouthwash.

At baseline, the MMP-8 level was different in the two groups (CG: 128.75 ± 30.74; SG: 82.26 ± 54.41), possibly due to the selection procedure that allowed to enroll patients with different stages of periodontitis. In order to compare the efficacy of the treatments, relative decrease was calculated at T1, T2 and T3 and the mean in differences between SG and CG showed a statistically significative (*p* < 0.001), greater relative reduction in SG at follow-up every time for the MMP-8 levels. Interestingly, the SG results showed that the ozonated olive oil mouthwash slowed the decrease of BoP index, and, at the beginning of treatment (T1), of PI index as well. Moreover, between-group differences in PPD and PI at T2 and T3 were insignificant.

The results of our study were in accordance with a very recent randomized clinical trial (RCT) by A.C. Nicolini et al. evaluated the effect of ozonated water in plaque formation and gingival inflammation and showed that ozonated water did not affect gingival inflammation and biofilm [26]. By contrast, a study aiming to compare the effect of ozonated oil and chlorhexidine gel applied thrice a day by massage on plaque-induced gingivitis showed that both the antimicrobial agents were effective in reducing PI and GI [27]. The difference between the two studies was the vehicle of ozone application: oil seemed to be more effective on inflammatory processes than water.

It is difficult to compare the results of the current study with data reported by other authors, since there are no data relating to the evaluation of salivary metalloproteinases in patients with periodontitis after the use of a mouthwash based on ozonated olive oil.

Ozone analgesic and anti-inflammatory effects are driven by different targets of action: (1) decreased production of inflammatory production and (2) inactivation of metabolic products that mediate pain by oxidation and by improving the vascular supply that leads to more oxygen availability to tissue and higher rate of toxic product elimination. Ozone improves tissue regeneration and speeds up wound healing [28]. Moreover, ozone is a negatively charged ion that counterbalances the acidic environment in infection [29].

A recent RCT compared ozone gel to systemic antibiotics after third molar surgery and it found that a significative reduction in pain, swelling and trismus occurred, with no adverse event [30].

Adverse events associated with mouthwashes use over time can include burning sensation/irritation/swelling/sensitivity in cheek, tongue, lips, gum, palate or papillae; tooth sensitivity; peeling/exfoliation/roughness in cheek, tongue, lips or gum; the presence of aphthous ulcer/wounds; itching/tingling/taste changes in cheek, tongue or lips; gastrointestinal signs and symptoms; and eye irritation. Chlorhexidine, the gold standard among the existing local antiseptic agents, presents with several side effects when used over time (tooth discoloration, taste alterations and oral desquamation) and the clinical outcomes may be hampered by lack of patient compliance [12,13,14]. None of the enrolled patients reported any of the listed adverse events over a duration of 3 moths.

An interesting study was conducted by Uitto et al. [31], according to which PMN cells that enter the mouth through the gingival sulcus are the main source of salivary collagenase. In addition, patients with periodontitis have shown the predominance of the active form of this enzyme, while healthy subjects have the latent form. MMP-8 activity and concentrations are under tight regulation to prevent extensive damage to connective tissue. Most MMPs are not continuously secreted into healthy tissues. MMP transcription requires the presence of some inflammatory mediators: interleukin-1β (IL-1β), tumor necrosis factor-alpha (TNF-α), transforming growth factor beta (TGF-β) and prostaglandin E2 (PGE2). Moreover, inflammatory markers and mediators such as c-reactive protein (CRP), interleukin-6 (IL-6) and TNF-α are present in systemic inflammation induced by hyperglycemia. In fact, a recent study aiming to investigate the association between serum glycosylated hemoglobin (HbA1c) levels and periodontal status in patients with periodontitis showed that the presence of periodontitis and tooth loss was negatively correlated with high serum HbA1c levels before diabetes onset. In addition, patients with periodontitis showed an increased risk for cardiovascular disease, stroke and metabolic syndrome [32].

In inflammation-based conditions, metabolism causes acidification of the microenvironment, with a subsequent condition called acidosis. Korostoff et al. [33] noted higher MMP-8 activity in slightly alkaline pH. Taking into consideration that a more neutral pH variation can be due to the elimination of acidogenic flora following the application of ozone, it can be assumed that ozone therapy optimizes the environment for greater MMP activity [34]. However, Bocci [35] pointed out that ozone therapy can progressively inhibit MMP secretion through a gradual reduction in plasma levels of platelet-activating factor (PAF), leukotriene B4 (LTB4), PGE2 and thromboxane A2 (TXA2). The author also stated that only long-term application, at least 6 months, of ozone can effectively delimit periodontitis. The problem of tissue hypoxia and impaired perfusion in chronic infection seems to be equally important. A study conducted by Polish researchers demonstrated the usefulness of ozone therapy in regulating blood supply to tissues [36].

A limitation of this study was the enrollment of patients with different stages of periodontitis, that resulted in baseline significant differences in study parameter means at baseline, except for PPD index. Moreover, the unexpected lack of effect of the ozonated olive oil mouthwash on periodontal indices may be due to the lack of compliance among the patients of the study group. Further RCTs to establish the effect of the ozonated olive oil mouthwash on periodontal indices in patients with chronic periodontitis are needed. It would be of interest to analyze the gender effects on periodontitis and on adjuncts in periodontal therapies.

## 5. Conclusions

Non-surgical periodontal treatment adjuvated by the use of ozonated oil led to a significant and faster reduction in saliva MMP-8 concentrations in patients with periodontitis. Periodontal indices decreased both in the study and control group, with no statistical significance.

## Figures and Tables

**Table 1:** Descriptive statistics in the study and control group.

Measurement	Index	Study Group		Control Group	
		Mean	SD	Mean	SD
T0	PI	56.75	25.95	77.33	21.10
	BoP	54.67	27.07	76.08	27.27
	PPD	4.426	1.391	4.394	0.781
	MMP	82.26	54.41	128.75	30.74
T1	PI	18.58	6.503	23.75	8.456
	BoP	20.83	8.270	27.50	13.31
	PPD	2.351	0.899	2.884	0.785
	MMP-8	63.12	48.82	115.9	30.04
T2	PI	10.25	8.170	14.83	5.684
	BoP	12.67	5.926	14.25	7.930
	PPD	2.15	0.922	2.206	0.781
	MMP-8	45.48	37.96	106.0	29.18
T3	PI	7.417	5.242	9.250	3.917
	BoP	7.000	3.377	8.250	4.190
	PPD	2.083	0.814	2.451	0.746
	MMP-8	32.33	27.76	94.32	26.91

PI (Plaque index); BoP (Bleeding on probing); PPD (Probing pocket depth); MMP-8 (metalloproteinase-8).

**Table 2:** Comparison of relative decreases of MMP-8 values and indices in the study group and control group.

Measurement	Outcome	Study Group		Control Group		Difference SG–CG		
		Mean	Std. Error	Mean	Std. Error	Mean	Std. Error	*p*-Value
T1	PI	0.6864	0.013	0.6313	0.019	**−0.0551**	**0.024**	**0.022**
	BoP	0.6221	0.023	0.5503	0.034	-0.0718	0.041	0.085
	PPD	0.3354	0.023	0.4181	0.037	0.0828	0.044	0.065
	MMP-8	0.1038	0.005	0.2867	0.015	**0.1829**	**0.016**	**<0.001**
T2	PI	0.8074	0.008	0.8115	0.022	0.0041	0.024	0.863
	BoP	0.7994	0.013	0.7342	0.022	**−0.0652**	**0.026**	**0.014**
	PPD	0.4853	0.028	0.4600	0.041	−0.0253	0.050	0.616
	MMP-8	0.1841	0.008	0.4992	0.016	**0.3151**	**0.018**	**<0.001**
T3	PI	0.8777	0.008	0.8692	0.016	−0.0084	0.018	0.640
	BoP	0.8868	0.010	0.8536	0.012	**−0.0331**	**0.016**	**0.040**
	PPD	0.4326	0.023	0.4652	0.040	0.0327	0.046	0.483
	MMP-8	0.2755	0.009	0.6509	0.014	**0.3755**	**0.016**	**<0.001**

Positive values of mean in difference SG–CG indicate greater relative reduction in SG, while negative values indicate greater relative reduction in CG. Statistically significant differences are presented in boldface.
